# Experiencias y transformaciones subjetivas: sufrimiento y coerción en el uso de psicofármacos en el tratamiento psiquiátrico en Chile

**DOI:** 10.18294/sc.2024.5388

**Published:** 2024-12-10

**Authors:** Manuel Alejandro Castro

**Affiliations:** 1 Doctor en Sociología. Académico, Departamento de Trabajo Social, Facultad de Ciencias Sociales, Universidad Alberto Hurtado, Santiago, Chile. alejandrocastroharrison@gmail.com Universidad Alberto Hurtado Departamento de Trabajo Social Facultad de Ciencias Sociales Universidad Alberto Hurtado Santiago Chile alejandrocastroharrison@gmail.com

**Keywords:** Salud Mental, Atención en Salud Mental, Psiquiatría, Psicofármaco, Chile, Mental Health, Mental Health Assistance, Psychiatry, Psychotropic Drugs, Chile

## Abstract

Este artículo analiza el impacto del uso de psicofármacos en personas diagnosticadas con esquizofrenia, trastorno afectivo bipolar y depresión severa en Chile. A través de un enfoque cualitativo narrativo, se recogen las experiencias de 25 pacientes entre los 2018-2021, quienes describen cómo estos medicamentos, aunque efectivos para controlar los síntomas, generan sufrimiento psíquico y una sensación de coerción en su vida diaria. Los resultados muestran que el uso prolongado de psicofármacos provoca efectos secundarios significativos, como el deterioro físico, cognitivo y social, y que los pacientes sienten una dependencia constante hacia estos medicamentos. Además, el artículo critica la medicalización de los trastornos mentales en el contexto de la psiquiatría moderna, donde predomina el enfoque biomédico, reduciendo el malestar humano a un problema neuroquímico y desestimando factores sociales. Se concluye que, aunque los psicofármacos pueden estabilizar a los pacientes, también perpetúan formas de control y sufrimiento.

## Abstract (en)

This article analyzes the impact of psychotropic drug use on individuals diagnosed with schizophrenia, bipolar affective disorder, and severe depression in Chile. Using a qualitative narrative approach, the experiences of 25 patients from 2018 to 2021 are examined. Participants describe how these medications, while effective in symptom control, generate psychological suffering and a sense of coercion in daily life. The results reveal that prolonged use of psychotropic drugs leads to significant side effects, including physical, cognitive, and social deterioration, as well as a persistent sense of dependence on these medications. Furthermore, the article critiques the medicalization of mental disorders within modern psychiatry, where the biomedical approach predominates, reducing human distress to a neurochemical problem and disregarding social factors. The study concludes that while psychotropic drugs can stabilize patients, they also perpetuate forms of control and suffering.

## INTRODUCCIÓN

El uso de los psicofármacos -tanto en Chile como en el mundo- se ha convertido en una realidad que se ha ido instalado progresivamente en las sociedades actuales, consolidándose como una de las principales intervenciones para la psiquiatría en el campo de la salud mental. Para Droguett^1^, se trata de un fenómeno creciente de tal magnitud que hoy en día se ha extendido incluso al mercado informal (ferias libres, mercados en línea, etc.), transformándose en un problema de salud pública. Hameed^2^ y Farooq^3^ coinciden en que el manejo correcto de los psicofármacos tiene un inmenso potencial de mejorar la calidad de vida; sin embargo, existe un peligro inminente a propósito de su uso inapropiado. Para Da Silva^4^, el alto consumo de psicofármacos en el mundo sería más característico de un problema de injusticia social, que medicaliza las conductas y emociones de las personas.

Estos fenómenos han ido avanzando sobre cuestiones impensables desde hace algunas décadas, tales como la medicalización de las emociones^5^; la psicofarmacologización de la tristeza^6^^,^^7^^,^^8^; la medicalización de la timidez^9^^,^^10^; la medicalización del amor^11^, o la grotesca medicalización de las personas en situación de calle^12^. De acuerdo a Elliot^13^, con el fin de buscar estabilidad y felicidad en la vida humana, antidepresivos como el Prozac (fluoxetina), o el descubrimiento de la clozapina, vinieron a revolucionar nuestra sociedad y reconvertirla. 

La cultura de la felicidad^14^ se convierte en una impronta en las sociedades actuales, y alcanzarla nos conduce a la paradoja de la vida plena. Sin embargo, cuando este propósito no se consigue comienza a desmoronarse esa virtualidad de bienestar, terminando en un derrotero, fracaso, o incluso en suicidio. Así, el pánico, el estrés, la depresión, los “trastornos de personalidad”, hasta las psicosis y los trastornos anímicos se transforman en respuestas a esos eventos. Tales consecuencias se plasman como una señal dolorosa y turbadora en las experiencias de los sujetos, como la sensación física de no controlar el propio cuerpo y los pensamientos. A pesar de todo, la promesa del bienestar recorre la cultura de las masas a través de la publicidad, las redes sociales, y todo medio de comunicación que *performan*^15^ los modos de ser al interior de una ideología de vida, estableciendo una cierta *doxa* del capitalismo^16^.

Este proceso llamado “medicalización”^17^^,^^18^^,^^19^^,^^20^ se ha ido empotrando en las sociedades del capitalismo tardío^14^, volviéndose en una nueva forma de control social, lo que implica un proceso en el cual los problemas humanos pasan a definirse y tratarse como problemas médicos. Involucra, por tanto, la aplicación de un modelo biomédico que considera la salud como la ausencia de enfermedad y se caracteriza por el reduccionismo, el individualismo y un sesgo hacia lo tecnológico^21^^,^^22^. No obstante, no se trataría de negar el derecho de la toma de antidepresivos o antipsicóticos, sino más bien a la medicalización excesiva como una señal de alarma^23^. De alguna forma esto también tendrá otras ramificaciones sociales como los sobrediagnósticos en salud mental, por ejemplo^24^.

De esta forma, el propósito de este artículo es mostrar cómo el uso y consumo de los psicofármacos se ha convertido en una realidad que ha transformado la vida de las personas con diagnósticos psiquiátricos en contextos de tratamientos públicos en salud mental, en Chile. A través de sus narraciones de vida se puede visualizar cómo esas experiencias han sido agenciadas por la psiquiatría y las prácticas gubernamentales de salud mental, moldeando sus vidas y construyendo ciertos tipos de personas sujetadas a tratamientos que han consolidado y gestionado el sufrimiento psíquico y social^25^^,^^26^.

### Psiquiatría y salud mental en tiempos neoliberales

Los psicofármacos como tecnología científica están percibidos, en la actualidad, como la gran solución individual a las enfermedades psíquicas. El consumo de estos artefactos ha venido creciendo exponencialmente en las sociedades del alto rendimiento^27^^,^^28^^,^^29^^,^^30^. De esta forma, los medicamentos psiquiátricos se han convertido en el discurso por excelencia de la salud mental y psiquiatría, siendo ampliamente utilizados en los distintos servicios de salud de todo el mundo, medicalizando la vida^31^. Este mecanismo tecnológico es central para explicar lo que se denomina paradigma biomédico, ideología dominante de la psiquiatría en el último tiempo^27^. En ese sentido, Moncrieff^32^ amplía la crítica hacia el paradigma biomédico al señalar que los medicamentos psiquiátricos han llegado a ocupar un rol central y casi exclusivo en los tratamientos de salud mental a nivel mundial. Esto refleja cómo la psiquiatría contemporánea ha abrazado una ideología donde la intervención farmacológica es vista no solo como la herramienta principal, sino como el núcleo del abordaje terapéutico. Según esta perspectiva, los fármacos no solo tratan síntomas, sino que también participan en la medicalización de la vida, es decir, en la interpretación y manejo de aspectos cotidianos y emocionales bajo parámetros médicos. Este enfoque limita la posibilidad de explorar otras formas de comprensión y tratamiento de los problemas de salud mental, reforzando un sistema que privilegia soluciones tecnológicas y farmacológicas sobre enfoques psicosociales o comunitarios. Moncrieff^32^ subraya, entonces, cómo este paradigma reduce la complejidad del sufrimiento humano a desequilibrios biológicos, marginando otras narrativas y estrategias de intervención.

El vínculo principal que ha tenido la psiquiatría y la salud mental con el modelo económico se ha dado a través de la industria farmacéutica^20^. Esta relación, muy conocida al interior de la salud en general, ha marcado una sinergia que incluso ha aprehendido a las políticas de salud mental en todo el mundo. De esa manera, se da inicio a la capacidad de gestionar el sufrimiento psíquico a través del mercado^33^. La industria farmacéutica -especialmente el desarrollo de la investigación relacionada con la medicina basada en la evidencia (MBE)- ha empujado a la psiquiatría a tomar un enfoque mucho más biológico para responder a la pregunta por las enfermedades mentales. De esta forma, “la psiquiatría biológica reduce la subjetividad del ser humano a lo que puede ser medible”^34^, es decir, todo se somete a interacciones neuronales y, por tanto, las respuestas a esos desequilibrios neuroquímicos -entendidos como “fallas” de procesos psicológicos y fisiológicos- serían solucionados por medio de intervenciones tecnológicas que suplen ese desequilibrio en el cerebro. Esto último se corregiría con el psicofármaco principalmente (entre otras terapias), que favorecerían que ese desequilibrio se estabilice. Ello falicitaría una “cosificación del sufrimiento”^34^, justificando la globalización del psicofármaco en todos los servicios de psiquiatría del orbe, en los que la industria farmacéutica cumple un rol esencial para la distribución del equilibrio psicopatológico, a través de los comprimidos para las enfermedades mentales.

El psicofármaco es entendido como el artefacto tecnológico por excelencia, que opera como instrumento fundamental de la psiquiatría y la salud mental en sus intervenciones clínico-sociales. El gasto en psicofármacos en EEUU al año 2001 era alrededor de 200 millones de dólares^35^, y para 2019 ese monto había crecido exponencialmente a 2.000 millones^36^. Para Angell^35^^),^ el objetivo de la industria farmacéutica no es desarrollar nuevos psicofármacos, sino más bien nuevos remedios lo suficientemente distintos como para extender nuevas patentes y conseguir introducirlas en el mercado. En el mundo de la salud mental esto es muy común, y el ejemplo más próximo lo encontramos en el antidepresivo denominado fluoxetina^37^^,^^38^.

La influencia de la industria farmacéutica a través de la psicofarmacología irá acompañada de una revolución silenciosa en la salud mental en el último cuarto del siglo XX. Los servicios psiquiátricos de todo el mundo se han vuelto dependientes de los medicamentos, a tal nivel que las intervenciones en este campo siempre se piensan estructuralmente desde la lógica farmacológica. Desde la invención de la clorpromazina como el primer antipsicótico, también llamado antipsicóticos de primera generación o atípicos, la dependencia entre la industria farmacéutica y la psiquiatría no se ha distanciado. Con la creación de la segunda generación de antipsicóticos, principalmente desde el descubrimiento de la clozapina en 1988, la psiquiatría y los departamentos de salud mental a nivel mundial han capitalizado los tratamientos que tienen referencia con las psicosis. De ese modo, las empresas farmacéuticas comenzaron a sintetizar nuevos psicofármacos derivados de la clozapina, tales como la olanzapina de Lilly o la risperidona de Janssen, antipsicóticos que hoy son de extendida comercialización y administración en los servicios de salud mental públicos y privados de todo el mundo. Así, la influencia de la industria farmacéutica y “las balas mágicas”, como las denomina Allen Frances^39^, comenzaron a influir directamente el mundo de la salud mental y la opinión pública, además de traer la solución a enfermedades que por más de cien años no se sabía cómo tratar, especialmente las psicosis. 

A lo anterior, se agrega el impacto que comenzaban a tener en la década de 1990 los psicofármacos en general en la vida cotidiana de las personas, traspasando el campo de la medicina psiquiátrica. Hasta antes de los psicofármacos, el tratamiento más común estaba relacionado con el encierro en las instituciones totales o manicomios (hoy llamados unidades de hospitalización psiquiátrica). Sin embargo, la introducción del psicofármaco en las sociedades postindustriales modificó el modo de entender la locura, ahora como un trastorno que puede ser atenuado, ya no solo con mecanismos coercitivos como el encierro, sino con una intervención neuroquímica que funciona silenciosamente en los cerebros de los seres humanos. Esto último se ve corroborado con el nacimiento de los antidepresivos y, en especial, con la influencia que el mercado tiene en la forma de disponer el arsenal farmacológico contra la depresión en los servicios de salud mental.

Si bien los primeros antidepresivos fueron comercializados a finales de la década de 1950, fue solo en la década de 1990 que se desplegó su uso masivo en un momento que muchos denominaron la era del “Prozac”^38^^,^^40^^,^^41^^,^^42^. El impacto social de la fluoxetina fue incluso mucho más importante que el descubrimiento de la clozapina y los antipsicóticos de segunda generación, ya que esta atacaba la nueva forma de la locura de las sociedades tardomodernas: la depresión.

Cuando la tercera versión del *Diagnostic and Statistical Manual of Mental Disorders* (DSM) de 1980^43^ introduce el enfoque basado en síntomas, comienza a proliferar en las sociedades contemporáneas la depresión como una nueva enfermedad que afecta a los seres humanos. El camino obligatorio que tenía esta nueva forma de entender el suicidio, la tristeza, el estrés o la melancolía, era el camino del psicofármaco. Tanto para Gøtzsche^38^ como para Frances^39^, la conexión entre los manuales diagnósticos y la industria farmacéutica es muy evidente en la forma de entender la depresión. Para Gøtzsche^37^^,^^38^, uno de los principales vínculos estaba en que miembros del comité de expertos del DSM-4^44^ eran personeros de las industrias farmacéuticas, algo que Allan Frances^39^ denunció en su momento. 

La era de la fluoxetina, que nace en la década de 1980 y se estabiliza en la de 1990, fundamentó el problema de la depresión, no como un problema social o consecuencia de las formas del vivir, sino más bien como síntomas de acuerdo con los manuales diagnósticos como el DSM-5^45^. El principal motivo señalaba al desequilibrio de los neurotransmisores, específicamente con la recaptación de la serotonina. Este descubrimiento, realizado por los científicos vinculados con la neuropsiquiatría, resultó revolucionario, ya que las causas de la depresión estaban asociadas a una “alteración de la función serotoninérgica y los ISRS [inhibidores de la recaptación de la serotonina] las restablecían, por lo que constituían un tratamiento específico y limpio de la depresión con muy escasos efectos secundarios”^42^.

El nacimiento de la fluoxetina nos proponía una nueva forma de intervenir el gran problema de la depresión. De la misma manera que ocurrió con la clozapina, se sintetizaron nuevos medicamentos con el principio de la fluoxetina y, más específicamente, con los ISRS tales como la venlafaxina, la sertralina o la paroxetina. Sin embargo, la fluoxetina es el antidepresivo que ha tenido más éxito en el mundo hasta la actualidad. A este fenómeno se lo podría denominar como la globalización del psicofármaco. Así, se calcula que, en el año 2010, el 5% de los hombres y el 10% de las mujeres en el mundo tomaban antidepresivos en los países de altos ingresos^46^. El uso de los antidepresivos, y especialmente el de la fluoxetina, se extendió de tal modo que fue colonizando a otras formas diagnósticas, tales como los trastornos de ansiedad, las fobias sociales, los trastornos obsesivos compulsivos, el tabaquismo, el estrés postraumático, el dolor crónico, los trastornos de personalidad, como también algunos casos específicos asociados al tratamiento auxiliar en las psicosis^47^. La era del Prozac se fue constituyendo lentamente como un momento trascendente en la nueva era de la psiquiatría. Las “pastillas de la felicidad”, como las denomina Nikolas Rose^48^, o las “balas mágicas”, llamadas así por Allan Frances^39^, influenciaron de tal modo la sociedad que los antidepresivos se convirtieron en un axioma conducente de la felicidad o, al menos, una ayuda para encontrarla. En ese sentido, Caponi^49^ plantea que la psiquiatría moderna, a través del uso de antidepresivos como el Prozac, ha contribuido a la construcción de una subjetividad humana influenciada por una biopolítica psiquiátrica. Esto significa que el uso masivo de estas “pastillas de la felicidad” no solo busca intervenir en la salud mental, sino que también configura modos de pensar, sentir y comportarse en sociedad. Al normalizar el consumo de antidepresivos como solución a problemas emocionales, se establece una narrativa en la que el bienestar emocional es gestionado por la medicina y los fármacos, moldeando a los individuos según ideales específicos de felicidad y productividad impuestos culturalmente. En este contexto, la biopolítica actúa como una herramienta de poder que regula y disciplina los cuerpos y las mentes a través de la medicina.

Así, los antipsicóticos, ansiolíticos, estabilizadores del ánimo y los antidepresivos se convirtieron en el bastión de las intervenciones psiquiátricas, en el mundo de la salud pública y privada. De esa manera, el malestar presente en las sociedades contemporáneas dejó de ser concebido como resultado de circunstancias adversas, desigualdades sociales, sobreexplotación o aumento del desempleo, y se vinculó con el padecimiento de una enfermedad y los desequilibrios de los neurotransmisores en el funcionamiento cerebral que, gracias a la ciencia y, especialmente a la psiquiatría y la industria farmacéutica, podían remediarse por medio de las “balas mágicas”^39^^,^^48^ que aseguraban la felicidad en el caso de la depresión, y la eliminación de las voces y desajustes conductuales, en el caso de las psicosis. 

Fue así como los expertos en salud mental se convirtieron en portavoces de las “buenas nuevas” que la psiquiatría trajo consigo en las sociedades del capitalismo tardío. En ese contexto, los profesionales de la salud mental -y especialmente los psiquiatras- se convirtieron en los voceros de la industria farmacéutica y de los discursos psiquiátricos de la nueva era del capitalismo.

Este tipo de discurso comenzó a proliferar en la psiquiatría desde la era del “Prozac”, hoy consolidado y expandida a nivel global, en el que la pregunta por la enfermedad mental se responde desde una elucidación científica, a propósito de desajustes neuronales, y no desde lo que implica un sufrimiento físico o social. Las personas, ahora convertidos en pacientes psiquiátricos, depresivos o esquizofrénicos, que intentaban contarnos sus historias, son reducidos a síntomas definidos por un manual diagnóstico que valida el raciocinio científico por sobre la experiencia subjetiva de las personas. En otras palabras, el significado interior de las personas que se aquejan por una tristeza, una separación o un sufrimiento social, se ve reducido a una interpretación científica atada al desequilibrio neuroquímico. 

El efecto global de estos discursos fue “transmitir a la población la idea que sin ayuda de expertos […] y sin el uso de tecnologías que el progreso había puesto a nuestra disposición, las gentes comunes iban a ser infelices”^42^. A esto último, se le suma la contribución de la Organización Mundial de la Salud (OMS) por transformar ese discurso en uno de tipo hegemónico. La difusión por parte de este organismo transnacional ayudó a darle un peso fundamental a los trastornos psiquiátricos. La salud en sí misma hoy es definida desde la propia salud mental: “Sin salud mental no hay salud”^49^. Este eslogan confirmaría la fuerte influencia que la psiquiatría tiene en nuestra época, convirtiendo un lenguaje biológico en uno social con capacidad performativa^50^. En este sentido, las predicciones de la OMS para el año 2030 afirman que la depresión será la principal causa de morbilidad a nivel mundial^51^, convirtiéndose en el mejor argumento de la globalización del psicofármaco y las implicancias económicas para la salud mundial.

### Chile y los psicofármacos

Esta influencia ha sido importante en Chile. Podemos encontrar algunos estudios que muestran el aumento sostenido del gasto público en psicofármacos. Una encuesta del año 2004 devela cómo el consumo en la Región Metropolitana alcanzaba alrededor del 6,4% de la población, y que una parte importante refería el uso de ansiolíticos y benzodiacepinas^52^. Con la inclusión de la esquizofrenia en las Garantías Explícitas de Salud (GES) a partir del año 2008, todas las personas con primer episodio de esquizofrenia recibieron tratamiento psicofarmacológico, y dentro de ese universo el 85% de las personas tomaban el antipsicótico llamado risperidona^53^. 

La Encuesta Nacional de Salud en Chile de los años 2009-2010^54^, indicó que el consumo de antidepresivos era de alrededor del 7,8% en la población chilena, así como también los psicolépticos (ansiolíticos, antipsicóticos, etc.) con un 5,6% correspondiente a la población total encuestada, siendo el clonazepan el psicofármaco más consumido por la sociedad chilena. Si bien Chile tiene un bajo presupuesto en salud mental con relación al gasto total en salud^55^, existe un aumento sostenido en el consumo de psicofármacos y, por tanto, un gasto público en salud. El estudio de Cea^56^ indica que el gasto público aproximado en psicofármacos habría ascendido, en el año 2017, a más de mil millones de pesos (1 millón de dólares), que implica un incremento entre el año 2011 y 2017 de más del 119%. De esta manera, para el 2021 los antidepresivos serían los psicofármacos más vendidos en nuestro país, fenómeno asociado al diagnóstico de depresión^57^^,^^58^.

## METODOLOGÍA

Este trabajo se enmarca en la investigación doctoral de sociología Los efectos performativos de la psiquiatría en la vida de las personas diagnosticadas psiquiátricas: el sufrimiento de la locura^27^. El trabajo tiene un carácter descriptivo-comprensivo con un enfoque cualitativo, en el que participaron 25 personas de ambos sexos diagnosticados psiquiátricamente (12 hombre y 13 mujeres), que pertenecían al sistema público de atención de salud mental chilena, específicamente de la Región Metropolitana. 

Los participantes de esta investigación recibieron los diagnósticos de esquizofrenia, trastorno afectivo bipolar y depresión severa, denominados como trastornos mayores con cobertura pública, llamada Garantías Explicitas de Salud^59^. Entre los criterios de selección de los participantes se estableció, en primer lugar, que tuvieran la confirmación diagnóstica de dos años; en segundo lugar, que fueran usuarios activos de tratamientos psiquiátricos de dispositivos de atención de la salud mental pública chilena, tanto en hospitales generales, hospitales psiquiátricos como también dispositivos comunitarios de salud mental. Por último, que fueran personas mayores de 18 años, estabilizados psicopatológicamente. Para la selección se utilizó la técnica de muestreo intencional por conveniencia. 

El análisis se realizó desde una perspectiva narrativa temática^60^^,^^61^ con el fin de recoger las experiencias de las personas que han sido diagnosticadas psiquiátricamente y usan cotidianamente los psicofármacos. Para ello, se realizaron entrevistas en profundidad^60^ para lograr comprender de una manera acabada la experiencia de estas personas en relación con sus vivencias como usuarios de la salud mental chilena en torno al psicofármaco. En ese sentido, una de las temáticas principales ha sido la experiencia en torno al sufrimiento psíquico que conlleva tener un diagnóstico relacionado con la salud mental, y su vínculo con el uso de psicofármacos como tratamiento psiquiátrico. 

El análisis de la información fue desarrollado a través de un enfoque narrativo, en donde las entrevistas fueron trabajadas a través de índices temáticos, y en un segundo momento codificados a través del *software* ATLAS.TI. Este análisis permitió dar cuenta en dos momentos sobre los discursos narrativos, permitiendo profundizar la experiencia del sujeto entrevistado.

La investigación se ajustó a los criterios elaborados por la Agencia Nacional de Investigación y Desarrollo de Chile (ANID). Se aplicó el consentimiento informado de acuerdo con la resolución del comité de ética R-431 de la Universidad Alberto Hurtado y el Comité Asesor de Ética de ANID del año 2019. Las personas entrevistadas participaron voluntariamente, luego de haber sido informadas de la confidencialidad y el anonimato, además del objetivo de la investigación. 

Por último, para efectos de este artículo, las transcripciones de las personas entrevistadas se codifican de manera numérica E1, E2, etc.

## LA EXPERIENCIA DEL PSICOFÁRMACO, SUFRIMIENTO Y EFECTOS ADVERSOS

En Chile, al emerger el diagnóstico psiquiátrico apareció, de manera inherente, el tratamiento psicofarmacológico, un dispositivo tecnológico que contiene la dosis adecuada de ciertos químicos necesarios para disminuir o mejorar síntomas de una enfermedad, que se generan por un desequilibrio neuroquímico en el cerebro^62^. Esta tecnología aparece como la máxima expresión de los tratamientos psiquiátricos hoy en día, ya que, sin ella, cualquier tratamiento perdería sentido.

El psicofármaco surge como un instrumento tecnológico para abordar las enfermedades mentales y las consecuencias que estas tienen en la vida cotidiana, que buscan moderar el comportamiento desviado, como también corregir las anomalías, ajustar al individuo, además de restaurar y mantener a las personas en circuitos normalizadores de la vida cotidiana^48^. De este modo, el psicofármaco -popularmente llamado “pastillas” o “remedios”- se transforma en un axioma que escolta a los individuos silenciosamente en la vida cotidiana de quienes han sido diagnosticados psiquiátricamente. Aquel acompañamiento mudo que realizan “las pastillas” también se convierten en una garantía para cualquier tratamiento en salud mental, no tan solo para adultos, sino también para niños y jóvenes^63^. En ese sentido, Fernández Liria^42^ nos dice que el discurso del psicofármaco habría calado profundamente en la sociedad, cimentando las lógicas de intervención de los servicios de salud mental sin mediar los efectos secundarios de estos.

Al ser consideradas como desordenes neurobiológicos, la depresión, la esquizofrenia y la bipolaridad (a pesar de que la naturaleza de cada una es distinta), pasaron a ser intervenidas médicamente por medio del dispositivo psicofarmacológico. En consecuencia, este tratamiento se convirtió en la intervención de primera línea para todo tipo de enfermedades mentales incluso para el “dolor crónico”^42^.

Así, el malestar humano, el dolor y el sufrimiento de las sociedades avanzadas pasa a ser concebido no como una respuesta a circunstancias adversas o el resultado de historias biográficas de sufrimiento o vivencias desafortunadas, “*sino como consecuencias de una enfermedad* […] *pero que gracias a los avances de la ciencia podía ser remediable*”^42^. Por tanto, la desgracia humana queda subsumida ante una explicación diagnóstica, e intervenida a través de una operación químico-tecnológica a largo plazo, en la que el psicofármaco es la herramienta principal para abordar la desdicha humana.

El psicofármaco se convierte en un poderoso instrumento para bajar la ansiedad, el dolor psíquico o la angustia. El sufrimiento psíquico^64^^,^^65^ tiene una salida limpia y eficaz a través de estos dispositivos tecnológicos que funcionan de modo eficaz, apagando el dolor que cursa un individuo cotidianamente. 

Muchas de las personas entrevistadas en esta investigación afirmaron que los psicofármacos eran parte de su vida, sin ellos no podían pensar su vida cotidiana, y que justamente servían para bajar la ansiedad, la tristeza, pero especialmente la descompensación: “*no sé, si dejara las pastillas yo creo que me descompensaría*” (E1), dijo una de las entrevistadas. En ese sentido, la toma de los psicofármacos se convierte en la columna vertebral de todo tratamiento psiquiátrico. 

Otro entrevistado se refirió a los psicofármacos “*como la base, ahí se empieza a levantar toda la estructura del tratamiento*” (E2), y, por otro lado, que evitaba la hospitalización. En este último caso, la toma de psicofármacos aparecía como una expresión para evitar el dolor de la hospitalización psiquiátrica: “*si me hubiera tomado las pastillas en la casa* […] *hubiera evitado la hospitalización*” (E14). De ese modo, la toma de psicofármacos que aparece como la estructura base del tratamiento psiquiátrico, emerge estratégicamente para evitar la descompensación, que para muchos tiene como consecuencia el encierro psiquiátrico: “*No quiero llegar a la hospitalización, por eso me tomo los remedios*” (E3).

Esta forma de coerción se edifica silenciosamente como un signo de dominio sobre el individuo afecto a un diagnóstico psiquiátrico, abarcando incluso a la familia, quien en muchos casos se hace cargo del tratamiento. Esta práctica se vive como una experiencia de autorresponsabilidad que vincula al individuo con una forma de vida que se reproduce cotidianamente: “*Yo sé que los medicamentos me hacen bien y todo eso* […] *y yo no quiero fallar, no fallarme a mí, no fallar a mi familia*” (E4). Por tanto, el tratamiento psiquiátrico, y en especial la toma de psicofármacos, se transforma en una manera de vivir, que exige al usuario de la salud mental autoimponerse una cierta norma de responsabilidad, una obligación a sí mismo, y que impida el sufrimiento, de manera de no generar dolor en quienes lo rodean, especialmente la familia, y así no cruzar la internación psiquiátrica: “*El tratamiento lo sigo sí o sí, nunca olvido tomarme los medicamentos. Los fármacos son el tratamiento, y actuar con responsabilidad y no andar haciendo tonteras, así no me encierran*” (E4).

En consecuencia, el tratamiento psicofarmacológico se establece como el modelo de intervención hegemónica en este campo. La representación simbólica de los remedios, llámese a estos antipsicóticos, estabilizadores del ánimo o antidepresivos, son el principio de cualquier tratamiento y, por ende, la forma primaria de intervención que la salud mental tiene en relación con las enfermedades mentales. De acuerdo a Múzquiz y Mata^28^ el psicofármaco colonizó la intervención de la salud mental, entregando una explicación biológica y no social en torno a las enfermedades mentales y, como consecuencia, todo diagnóstico psiquiátrico debe psicomedicalizarse.

Las narrativas en torno al uso del psicofármaco corroboran las ideas de los autores mencionados: “*Así que me dio unos remedios porque le dije que andaba como sin ánimo, pero nunca supe las pastillas que me dio*” (E1). Asimismo, otros entrevistados decían:

“*Me llenaban de pastillas, pero no tenía ningún avance y yo seguía sintiendo eso, y no volví a dormir naturalmente hasta ahora*”. (E2)

“*Un día el psiquiatra me preguntó tres cosas y me dijo: no, usted es una persona bipolar, y debe tomar litio y lamotrigina, para siempre y nada más*”. (E20)

“*Estaba triste y me acerqué a Atención Primaria en busca de ayuda e inmediatamente me dieron un antidepresivo*”. (E6)

Las experiencias que emergen en torno al uso del psicofármaco en la salud mental responden a un modelo de intervención centrado en la acción del fármaco^28^. Por ello, cuando las personas relatan que el remedio es el tratamiento en sí, no es más que la construcción que la misma psiquiatría ha erigido como forma de intervenir. La noción de adherencia al tratamiento psicofarmacológico^66^ se ve arropada con otras intervenciones de índole psicosocial secundarias (talleres, terapias, psicoeducación, etc.) que favorecen la impresión de que el fármaco es lo principal en un tratamiento de salud mental. Esto se expresa en lo que decía el siguiente usuario: “*Yo estoy acostumbrada a tomar remedios, tengo mis horarios, mis pastilleros, entonces los hice parte de mi vida cotidiana*” (E8).

El psicofármaco está relacionado como una estrategia de afrontamiento frente al sufrimiento. Lo interesante es que toda persona que se enfrenta a un diagnóstico psiquiátrico nunca más puede escapar del tratamiento psicofarmacológico, ya que una vez que “se llega al diagnóstico, resulta muy difícil revertirlo; se queda allí para siempre”^38^. Esto se expresa en lo que indicaba una de las personas entrevistadas:

“*Sin los remedios estaría rodando en mi vida, si no fuera adicto a la farmacia no sé que sería de mí. Es que el fármaco, después de tomarlo por 30 años, es parte de mi vida. Si me levanto el remedio este ahí́, si voy al baño, si voy al hospital, si voy a comprar pan, es decir en todos lados voy con mis remedios, es algo casi natural, lo hago por instinto. Si alguna vez se me llega a olvidar es fatal, pero me hacen bien*. […]. *Sin los remedios no podría dormir y no podría funcionar, estoy tan acostumbrado a ellos, y llevo tantos años, yo creo que me haría mal si no me tomara mis remedios, y bueno, obvio, si no me los tomo me internan*”. (E9)

### Efectos secundarios de los psicofármacos y el sufrimiento

El tratamiento psicofarmacológico en psiquiatría se convierte en una tecnología de largo alcance que suscita periodos prolongados en su uso y que, como cualquier fármaco, tiene efectos secundarios^41^. A propósito de lo anterior, uno de los entrevistados manifestaba dicha preocupación:

“*Esas pastillitas me las empezaron a dar desde joven, y comencé a tomarlas sin mayor conciencia de lo que eran. En ese tiempo, uno se tomaba lo que el psiquiatra le decía que tomara, y uno no cuestionaba eso, así que comencé a tomar fármacos desde los 16 años, y ahora llevo tomando como 40 años remedios. Con pastillas, es la única forma que conozco de vivir. Vivir esta enfermedad es vivir tomando remedios, y sabes que te dañan, mira como soy a los 56 años, parezco de 70, y son los remedios, todo para que no sea un peligroso. Ahora, pienso lento, me cuesta caminar, tengo temblores, me hacen exámenes todos los meses, vivo en un hogar protegido, me manejan los remedios, etc. Es una prisión, que debo aceptar porque es mejor vivir con fármacos que encerrado en un psiquiátrico*”. (E12)

Esta narración no sería aislada. Otras personas usuarias de la psiquiatría chilena diagnosticadas con depresión indicaban el malestar singular que le provocaban algunos fármacos:

“*El antidepresivo que estaba tomando, no me acuerdo bien, pero creo que era la sertralina, pero también fue con la fluoxetina, cuando los empecé a tomar me quedaba dormido en todos lados, y empezaron todos los síntomas que uno comienza a tener cuando uno toma antidepresivos: náusea, diarrea, me costaba concentrarme, etc., a mi mamá no le gustó, y me dijo: ‘yo no te voy a estar cuidando tu hijo mientras tú duermes o estás tirada en la cama’. Entonces me conflictué y preferí dejar de tomar los fármacos porque debía cuidar a mi hijo*”. (E7)

Para muchas personas, los efectos secundarios son una causa más de estigmas^41^, provocando una aflicción adicional a la propia enfermedad diagnosticada. Esto se corrobora con lo que una usuaria con depresión manifestaba: “*es molesto, porque tengo salivación, me hace parecer tonta, y en este caso es importante para mí, imagínese si me ve así mi familia, piensan que soy una tonta*” (E8). De esa manera, se incrementa mucho más la angustia y el sufrimiento de las personas que cursan tratamientos psicofarmacológicos frente a situaciones estresantes. En esa línea, otro usuario refería la paradoja que significaba el uso prolongado de esta tecnología: “*Los fármacos me han servido mucho, aun así, tomo 600 miligramos de clozapina, que es mucho creo yo, y hablo como ebria. Los efectos secundarios de la clozapina son horribles*” (E19).

De esta manera, las personas participantes de esta investigación relataban su experiencia al usar longitudinalmente los psicofármacos y las dificultades que tenían con los profesionales médicos: “*Yo les decía a los médicos que las pastillas me tenían inquieto, porque yo no era así, además no podía dormir, porque movía mi boca para todos lados, y, aun así, no me quitó los remedios*” (E2). 

Así mismo, otro usuario refiere respecto al uso de antipsicóticos:

“*La clozapina que tomo me afecta para las cosas más diminutas y simples, no puedo ni escribir ahora porque me tiembla mi mano, y eso me da vergüenza porque yo era el mejor del curso, y ahora no puedo ni escribir y menos aprender a manejar un auto* […] *desde que tomo fármacos siento esa sensación, este temblor. Ahora, tomar una sopa con cuchara me es difícil, por eso me aburre el tratamiento, pero sé que tengo que seguir viniendo, sino será́ peor para mí. Pero creo que los remedios arruinaron mi vida*”. (E16).

Los efectos secundarios, que muchas veces perduran en la vida de las personas, abaten la vida de sus consumidores, profundizando su dolor subjetivo. Y, en ese sentido, no solo la clozapina, como antipsicótico tendrá este efecto, también podrá verse con la olanzapina:

“*Cuando estaba con el doctor, este me daba olanzapina, al principio me daban 5 miligramos o algo así, y ahí me sentía mal, cuando me tomaba la pastilla, más tarde estaba con las piernas inquietas, como que se me movían solas, no podía estar ni acostada y menos dormir. Después, me daban una de diez miligramos y ahí fue peor, entonces recuerdo que me daban más fármacos para que los otros remedios apagaran los síntomas de la olanzapina, y yo le decía al médico que me cambiara el remedio, pero él no me escuchaba, decía que era por mi bien*”. (E15)

Lo anterior también puede verse con otro usuario:

“*La risperidona y las benzodiacepinas que tomaba me tenían muy mal, además de darme mucho sueño, veía borroso, como si fuera miope. Además, era como un zombie, era caminar sin pensar y como desvanecido, le explicaba al médico esto, pero me decía que era lo que debía pasar, y eso me angustiaba más*” (E4).

La dicotomía que produce el consumo de psicofármacos, por un bien mayor, genera una profunda escisión emocional en las personas usuarias, pocas veces abordadas, causando resignación, coerción y sufrimiento.

“*Los remedios han tenido un efecto malo y bueno en mi vida. A mí no me gustan, me mantienen aturdido, pero no saco nada con decirle a algunos médicos, porque lo que a ellos le importa es que uno no se descompense. La verdad, no le creen mucho a uno, yo pienso que creen que es la enfermedad la que a uno lo mantiene así, pero uno sabe que son las pastillas. A pesar de ello, igual confío en los médicos, sino ¿en quién?*”. (E5)

## DISCUSIÓN

Para el investigador clínico Peter Gøtzsche, los diagnósticos son tan peligrosos como los nuevos fármacos^38^ y, en ese sentido, para este autor la explosión de diagnósticos psíquicos en los últimos 30 años y el elevado consumo de psicofármacos asociados responde a la instalación técnico-política que ha desarrollado la psiquiatría en el mundo. En esa misma línea, Moncrieff^32^ nos dice que la influencia de la industria farmacéutica ha moldeado la percepción pública y profesional de los trastornos mentales como enfermedades médicas, promoviendo una dependencia excesiva a los psicofármacos, performando la percepción sobre este dispositivo técnico-clínico, como la gran solución a las enfermedades mentales, sin tener consideración en los efectos psicoactivos, secundarios y a largo plazo, además del impacto en la experiencia de los seres humanos.

De ese modo, la proliferación del consumo de psicofármacos en las sociedades contemporáneas tiene un efecto innegable, pero lo que no se dice abiertamente son sus consecuencias sobre las personas, algo que la medicina psiquiátrica denomina efectos secundarios^37^. El resultado que pueden tener los psicofármacos en las personas podría inducir a interpretaciones erróneas, como diagnosticar nuevos trastornos en las personas que consumen estos remedios. Todos los antipsicóticos, ansiolíticos, antidepresivos y estabilizadores del ánimo tienen efectos adversos alarmantes a largo plazo, pudiendo convertirse en una amenaza para la salud y, en algunas ocasiones, causar la muerte^37^^,^^38^^,^^39^^,^^41^^,^^47^. Ejemplo de ello son los efectos extrapiramidales, que implican una alteración a una red de neuronas relacionadas con la coordinación y los movimientos, siendo característicos del consumo de psicofármacos, especialmente de los antipsicóticos. 

El parkinsonismo, por ejemplo, es uno de les efectos extrapiramidales más comunes del uso de psicofármacos^41^, ya que, por ejemplo, la rigidez y los temblores del parkinsonismo son característicos del uso de antipsicóticos de primera y segunda generación. Por otro lado, las distonías (que son movimientos musculares involuntarios), o la acatisia (un tipo de agitación con inquietud extrema que desarrolla movimientos incesantes), además de las disquinesias (especialmente la tardía, que desarrolla movimientos involuntarios, normalmente de la mandíbula, la boca y la lengua) tienen efectos físicos directos en las personas que, al ser diagnosticadas psiquiátricamente, deben usar antipsicóticos por periodos prolongados. 

Lo que sienten los pacientes es una psicofarmacologización de su vida que, en palabras de Gøtzsche, “son los efectos predominantemente subjetivos que describen en Internet los enfermos cuando toman antipsicóticos, generalmente estos son sedación, déficit cognitivo, aplanamiento emocional e indiferencia”^38^. Sin embargo, los psicofármacos se promocionan como un gran avance médico, “ya que hacía que los enfermos se volvieran dóciles y callados, algo que los trabajadores psiquiátricos valoraban”^38^. Así, este equilibrio químico forzoso en las personas que tienen enfermedades psiquiátricas produce consecuencias nefastas para las experiencias de estos sujetos: como la adicción a los psicofármacos, uno de los secretos mejores guardados por la psiquiatría^32^^,^^37^^,^^38^^,^^66^^,^^67^.

La interacción de los psicofármacos con los receptores del cerebro influye en los neurotransmisores, pues tiene un efecto bioquímico similar a un “supresión de las reacciones emocionales o un atontamiento para que no preste tanta atención al empeoramiento de su calidad de vida”^38^. Por otro lado, esta tecnología, al igual que otras sustancias adictivas, alteran considerablemente la personalidad, dificultando la capacidad de llevar una vida con más normalidad, logrando que en muchos casos las personas terminen aisladas de la sociedad, lo que tiene un efecto contrario al que se suponían. 

El efecto *zombie* de los psicofármacos genera en las personas dificultades que se expresan en la vida diaria. Este andar como *zombie* -como refieren algunas personas- representa una analogía de caminar hacia cualquier lado sin conciencia de nada, sin pensar ni razonar. 

Las mismas personas reconocen que el psicofármaco tiene efectos complejos en su vida, y desde su propia experiencia distinguen cuándo es el fármaco y cuándo es la enfermedad. Así, los efectos secundarios tienen también consecuencias en la vida cotidiana de quienes usan los “remedios”, generando angustia y sufrimiento, ya que, por un lado, se sienten responsables de tomar estos medicamentos (frente a la decisión de un médico en relación con una enfermedad) y, por otro lado, se ven enfrentados a problemas cotidianos que les producen contradicciones y obligan a tomar decisiones que, a mediano o corto plazo, terminan interfiriendo en sus relaciones sociales. 

En definitiva, los efectos secundarios de los psicofármacos se constituyen como una experiencia de sufrimiento y coerción. Si bien estas no son evidentes o no se hacen patentes en todas las personas, gran parte de las personas entrevistadas en esta investigación manifestaron una relación conflictiva con el psicofármaco. El efecto secundario de las “balas mágicas de la psiquiatría” -como diría Whitaker^47^-, y esa “comorbilidad oculta” a la que Bentall^41^ hace referencia, se establecen como un elemento complejo que se ubica en la experiencia de la persona, sin que este sujeto pueda escaparse de dicha tecnología médica.

## CONCLUSIONES

Cuando hablamos sobre los efectos de los psicofármacos en la experiencia de las personas que cursan diagnósticos psiquiátricos, podemos ver cómo estos cambian radicalmente sus vidas, para bien o mal. De alguna manera los relatos de este trabajo nos pueden acercar a ese cambio de realidad, y lo ciertamente interesante es cómo esto se dio por medio de una tecnología psiquiátrica que transformó la vida de la persona usuaria de la salud mental. Uno de esos cambios radicales fue cómo el sufrimiento y la coerción fueron instalándose paulatinamente en la vida de estas personas, a tal punto que les resulta imposible cambiarlas una vez agenciados por la psiquiatría. 

El psicofármaco también ha tenido un efecto radical y performativo en la vida cotidiana de estas personas. Si bien, la internación involuntaria se ha convertido en una herramienta psiquiátrica que ha perdurado en el tiempo, desde la masificación del psicofármaco en la década de 1990, este se ha convertido en otra herramienta efectiva para poder controlar a quienes cursan tratamientos. La coerción psiquiátrica también se operativiza a partir de la administración de remedios. El psicofármaco construye un efecto altamente potente en la realidad del ser humano y especialmente en pacientes de la salud mental. Si bien muchos declaran que los remedios son buenos, y en muchas ocasiones ayudan a disminuir los síntomas como las voces, la tristeza vital y estabilizar el ánimo, en otras, se vuelven una cárcel, ya que tomar “para siempre” se convierte en una atadura que condiciona la vida de las personas. Más allá del juicio valórico que pueda significar tomar remedios, lo más importante en este sentido es cómo se ha interiorizado este artefacto en la vida de las personas, especialmente quienes tienen diagnósticos psiquiátricos. Es muy común que el “remedio” se visualice como un axioma en la vida cotidiana de las personas, y que se naturalice en su interacción social. Sin embargo, la violencia simbólica (y en otros física) que tiene el psicofármaco, condiciona y transforma la vida de los sujetos a quienes se le administran: “*en la mañana lo primero que hago son tomarme los psicofármacos, estoy tan acostumbrado que ya no sé qué pensar, solo lo hago*” (E14) y, como consecuencia, esta práctica inscrita en el valor de la salud, se convierte en una regla de vida, moldeando la conducta de un sujeto.

Las consecuencias que se imprimen en los cuerpos y las emociones de las personas, que por años llevan usando los psicofármacos, de algún u otro modo transforman y moldean, controlan y dirigen a la persona usuaria de la salud mental a una sumisión, ya que no hay otro camino -al menos en el imaginario social-, que pueda servir para amainar el sufrimiento psíquico generado por la enfermedad psiquiátrica. Tal como plantean otros autores^68^ las vivencias sobre el uso del psicofármaco transforma simbólicamente la vida, desde el conflicto con estos dispositivos tecnológicos, hasta una dimension emocional y motivacional^68^. Se podría hablar de un encadenamiento al psicofármaco, ya que por mucho que se quiera liberar de él, el sujeto no puede romper esa barrera que se mantiene arraigada en las explicaciones científicas, y que se han introducido como definiciones sólidas en relación con el tratamiento de enfermedades mentales.

Abandonar los psicofármacos trae consigo ciertas premisas de las que muchas veces el usuario de la salud mental es parcialmente consciente, lo cual no tiene que ver con el hecho de tener una enfermedad. Estos pueden ir desde las experiencias negativas asociadas al consumo prolongado, hasta las barreras institucionales^69^. Y, en ese sentido, los efectos secundarios s que tienen los psicofármacos, en términos biológicos y metabólicos en el cuerpo humano, son tremendamente importantes; no obstante, las explicaciones técnicas de la psicofarmacología no son necesariamente tan transparentes, además que tanto médicos como profesionales de la salud mental no explican todos los efectos secundarios que se pueden tener, dado que las consecuencias de años tomando psicofármacos son desastrosas para el cuerpo de las personas. 

Este trabajo tiene muchos relatos sobre esta cuestión, y estos van variando desde efectos tales como síndromes neurolépticos malignos, hasta los temblores. Lo que importa de fondo es que el psicofármaco si bien ha sido un artefacto científico y biopolítico de los más importantes para la psiquiatría y la salud mental, también se convierte en un dispositivo siniestro que, asociado al bien mayor, éticamente hablando, busca un control conductual de las personas afectas a diagnósticos psíquicos, y las consecuencias negativas en quienes lo usan a largo plazo quedan relegadas. Asimismo, los efectos que instituye el psicofármaco en los cuerpos y en las emociones, en muchas ocasiones, no están tan presentes en quienes los usan. Sin embargo, representan un cierto malestar subjetivo que se deja entrever como una forma de violencia de tipo simbólica, que moldea esa realidad humana.

## References

[1] Droguett N, Vidal C, Medina B, Hoffmeister L (2019). Factores asociados al consumo de psicofármacos sin receta en Chile: Estudio descriptivo basado en la Encuesta Nacional de Consumo de Drogas en la Población en General. Medwave.

[2] Hameed S (2019). Medicalization: A Growing Problem. Journal of the Scientific Society.

[3] Farooq UM, Sadiq S, Azam F (2021). Cover-Medicalization: A Modern Problem Divisible from Medicalization. Journal of Research in Medical and Dental Science.

[4] Neto HSM (2019). Let’s Play (Un)Medicalization?. Revista Praxis Educacional.

[5] Wechuli Y (2023). Medicalizing disabled people’s emotions symptom of a dis/ableist society. Frontiers in Sociology.

[6] Horwitz A, Wakefield J (2007). The loss of sadness: How psychiatry transformed normal sorrow into depressive disorder.

[7] Wakefield J (2012). Should prolonged grief be reclassified as a mental disorder in DSM-5? Reconsidering the empirical and conceptual arguments for complicated grief disorder. The Journal of Nervous and Mental Disease.

[8] van Dijk EL, van Tol D, Diemers A, Wienen A, Bastra L (2022). Sick or Sad? A qualitative study on how dutch GPs deal with sadness complaints among young adults. Frontiers in Sociology.

[9] Scott S (2006). The medicalisation of shyness: From social misfits to social fitness. Sociology of Health & Illness.

[10] Aho K (2010). The psychopathology of America Shyness: A hermeneutic reading. Journal for the Theory Social Behaviour.

[11] Earp BD, Sandberg A, Savulescu J (2014). The medicalization of love. Cambridge Quarterly of Healthcare Ethics.

[12] Aldeia J (2019). The grotesque medicalization of homelessness. Pensando-Revista de Filosofía.

[13] Elliott C, Parens E (1998). Enhancing human traits: Ethical and social implications.

[14] Berardi F (2015). La fábrica de la infelicidad: Nuevas formas de trabajo y movimiento global.

[15] Callon M (1998). The law of the markets.

[16] Boltansky L, Chiapello E (2002). El nuevo espíritu del capitalismo.

[17] llich I (2010). Limits to Medicine: Medical Nemesis: The Expropriation of Health.

[18] Conrad P (2007). The medicalization of society: On the transformation of human conditions into treatable disorders.

[19] Davis J (2010). Medicalization, social control, and the relief of suffering. En: Cockerham WC, (ed). The New Blackwell companion to medical sociology.

[20] Davies J (2022). Sedados: Cómo el capitalismo moderno creó la crisis de la Salud Mental.

[21] Clark J (2014). Medicalization of global health 1: Has the global health agenda become too medicalized?. Global Health Action.

[22] Svenaeus F (2023). The phenomenology of objectification in and through medical practice and technology development. The Journal of Medicine and Philosophy: A Forum for Bioethics and Philosophy of Medicine.

[23] Kaczmarek E (2019). How to distinguish medicalization from over-medicalization?. Medicine Health Care and Philosophy.

[24] Hofmann B (2016). Medicalization and overdiagnosis: Different but Alike. Medicine, Health Care and Philosophy.

[25] Castro MA (2020). El Sufrimiento Psíquico de las personas con un diagnóstico psiquiátrico, el dolor de la Locura. Revista Perspectivas.

[26] Castro MA (2021). Los efectos performativos de la psiquiatría en la vida de las personas diagnosticadas psiquiátricas: el sufrimiento de la locura.

[27] Múzquiz Jiménez Á, De la Mata Ruiz I, Desviat M, Pérez-Moreno A (2021). Acciones de la salud mental en la comunidad.

[28] Silva M, Antunes A, Azeredo-Lopes S, Cardoso G, Xavier M, Saraceno B, Caldas de Almeida J (2020). How did the use of psycotropic drug change during the Great Ressecion in Portugal? I follow-up to the National Mental Health Survey. BCM Psychiatry.

[29] Oliveira JRF, Varallo FR, Jirón M, Ferreira IML, Siani-Morello MR, Lopes VD, Leira L (2021). Descripción del consumo de psicofármacos en la atención primaria en salud de Riberäo Preto en estado de Sao Paulo. Cadernos de Saúde Pública.

[30] Villalobos-Madriz J, Arias-Serrano B, Chacón-Arguedas S, Závaleta-Monestel E, Miranda-Rodríguez R, Fernández-Cheverri J, Gómez-Covarrubias A (2023). Prescribing trends in psychotropic medications among outpatients of a Latin American healthcare setting: A five-year retrospective study. Cureus.

[31] Martinez M (2023). The medicalization of life: an interdisciplinary approach. Helyon.

[32] Moncrieff J (2013). Hablando Claro, una introducción a los fármacos psiquiátricos.

[33] Castro MA (2019). Salud mental y gubernamentalidad: Reflexiones en torno a la locura en Chile. De Prácticas y Discursos.

[34] Lobo-Ortiz A, Huertas R (2018). Críticas y alternativas en psiquiatría.

[35] Angell M (2004). The truth about drug companies: How they deceive us and what to do about it.

[36] Dorahy G, Chen JZ, Balle T (2023). Computer-aided drug design towards new psychotropic and neurological drugs. Molecules.

[37] Gøtszche P (2015). Medicamentos que Matan y Crimen Organizado.

[38] Gøtszche P (2016). Psicofármacos que Matan y Denegación Organizada.

[39] Allen F (2014). ¿Somos todos enfermos mentales? Manifiesto contra los abusos de la psiquiatría.

[40] Wurtzel E (2001). Nación Prozac.

[41] Bentall R (2011). Medicalizar la Mente: ¿sirven de algo los tratamientos psiquiátricos?.

[42] Fernández Liria A (2018). Locura de la Psiquiatría, apuntes para una crítica de la psiquiatría y la “salud mental”.

[43] Asociación Americana de Psiquiatría (1988). Manual de diagnóstico y estadística de trastornos mentales.

[44] Asociación Americana de Psiquiatría (1994). Manual de diagnóstico y estadística de trastornos mentales.

[45] Asociación Americana de Psiquiatría (2013). Manual de diagnóstico y estadística de trastornos mentales.

[46] Organization for Economic Co-operation and Development (OECD) (2015). Health at a Glace 2015: OECD Indicators.

[47] Whitaker R (2011). Anatomía de una Epidemia, Medicamentos Psiquiátricos y el asombroso aumento de las Enfermedades Mentales.

[48] Rose N (2012). Políticas de la Vida: Biomedicina, poder y subjetividad en el siglo XXI.

[49] Caponi S (2023). Política, Psicofármacos y Vida Cotidiana.

[50] Organización Mundial de la Salud (2004). Promoción de la Salud Mental, conceptos, evidencia y práctica.

[51] Ramos C (2012). El Ensamblaje de Ciencia social y sociedad, conocimiento científico, gobierno de las conductas y producción de lo social.

[52] Organización Mundial de la Salud (2011). Carga mundial de trastornos mentales y necesidad de que el sector de salud y el sector social respondan de modo integral y coordinado a escala país.

[53] Rojas G, Fritsch R, Galleguillos T, Gaete J, Araya B (2004). Consumo de psicofármacos en la población general del Gran Santiago, Chile. Revista de Psiquiatría Clínica.

[54] Alvarado R, Minoletti A, Markkula N (2009). Adherence to Guidelines and Treatment Compliance in the Chilean National Program for First-Episode Schizophrenia. Psychiatric Services.

[55] Gobierno de Chile, Ministerio de Salud (2010). Encuesta Nacional de Salud ENS 2009-2010: Informa Final.

[56] Errázuriz P, Valdés C, Vöhringer PA, Calvo E (2015). Financiamiento de la Salud Mental en Chile: una deuda pendiente. Revista Médica de Chile.

[57] Madrid-Cea JC (2018). Estado neoliberal y gasto público en psicofármacos en el Chile Contemporáneo. Santiago: Revista de Psicología, Conocimiento y Sociedad.

[58] Gobierno de Chile, Ministerio de Salud (2021). Departamento Economía de la Salud. Análisis sobre el mercado de medicamentos en Chile en consumo y ventas a nivel de ATC parala serie disponible 2011-2020 y comparación con países de la OCDE.

[59] Gobierno de Chile, Ministerio de Salud, Departamento Economía de la Salud (2022). Análisis de los medicamentos incluidos en las Garantías Explicitas de Salud.

[60] Gobierno de Chile, Ministerio de Salud (2013). Guía Clínica AUGE: Depresión en personas de 15 años y más.

[61] Kohler-Riessman C (1993). Narratives Analysis.

[62] Chase S, Denzin N, Lincoln Y (2015). Métodos de recolección y análisis de datos, Manual de Investigación Cualitativa, vol IV.

[63] Rose N (2019). Our psychiatric future, the politics of mental health.

[64] Coelho L, Neves T (2023). Psychic suffering in neoliberalism and the political dimension of the mental health diagnosis. Saúde e Sociedade.

[65] Castro MA (2023). Coerción en las hospitalizaciones psiquiátricas en Chile: el sufrimiento de la locura en el siglo XXI. Salud Colectiva.

[66] García-Laborda A, Desviat M, Moreno A (2012). Acciones de la Salud Mental en la Comunidad.

[67] Read J, Mosher LR, Bentall RP (2006). Modelos de Locura: Aproximaciones psicológicas, sociales y biológicas a la esquizofrenia.

[68] Martínez-Granados F, Briones-Vozmediano E, Ronda E (2024). Vivir con psicofármacos: un estudio fotovoz comunitario en personas con alta adherencia al tratamiento en el sureste de España. Salud Colectiva.

[69] Castillo Parada T (2018). Subjetividad y autonomía: signifi cados y narrativas sobre la discontinuación de fármacos psiquiátricos. Salud Colectiva.

